# HMGB1 and injury amplification

**DOI:** 10.18632/oncotarget.5243

**Published:** 2015-08-22

**Authors:** Peter Huebener, Celine Hernandez, Robert F. Schwabe

**Affiliations:** Department of Medicine, Columbia University, New York, NY, USA

**Keywords:** HMGB1, RAGE, DAMPs, inflammation, cell death

While apoptotic cell death is believed to be mostly non-reactive and hence a component of development and tissue homeostasis, other forms of cell death such as necrosis and necroptosis are considered reactive, resulting in strong inflammatory responses. In contrast to infection, where non-self molecular signatures trigger immune responses, the mechanisms that trigger inflammatory responses to sterile tissue injury remain ill-characterized. It has been proposed that specific molecules termed damage-associated molecular patterns (DAMPs - also called alarmins) activate an injury sensing system that in many ways resembles the activation of innate immunity by pathogen-associated molecular patterns (PAMPs). Understanding signals that trigger inflammation following tissue injury is highly relevant as overshooting inflammation in this setting can be deleterious. Hence, targeting DAMPs or their receptors may open up novel therapeutic opportunities for a wide range of diseases.

The liver represents an excellent model system to study responses to cell death. Because of its central role in metabolism and detoxification, the liver is inherently at high risk for life-threatening toxic injury. One classic example is acetaminophen intoxication, which leads to massive liver necrosis and constitutes the leading cause of acute liver failure. Toxic liver injury typically triggers necrotic cell death, which is reflected by profound release of DAMPs including high mobility group box 1 (HMGB1). Moreover, chronic cell death is a key disease driver in the liver, making it an ideal model to examine the role of DAMPs in chronic diseases such as fibrosis and cancer [1].

As global deletion of *Hmgb1* results in early postnatal lethality [2], we addressed its role in sterile inflammation in a new model of conditional *Hmgb1* deletion [3]. Mice with hepatocyte-specific *Hmgb1* deletion displayed normal viability without any abnormalities at baseline or under conditions of metabolic stress [3]; however, when subjected to liver necrosis induced by acetaminophen overdose or ischemia/reperfusion injury, HMGB1-deficient mice displayed significantly reduced inflammation and neutrophil infiltration despite similar initial injury compared to their wild-type counterparts [4]. Similar findings were made in mice lacking HMGB1 receptor RAGE on bone marrow-derived cells, but not in mice lacking TLR4, another HMGB1 candidate receptor. Hence, HMGB1- RAGE provides an important molecular link between cell death and subsequent sterile inflammation. Importantly, HMGB1-mediated inflammation was only detected in the setting of tissue necrosis, as there was no effect of HMGB1 deficiency in models of Fas- and TNF-induced hepatocyte apoptosis. Of note, inactivation of HMGB1 or RAGE as well as genetic inhibition of neutrophil activation resulted in profound reduction of liver injury at later time points, suggesting a HMGB1- and RAGE-dependent and neutrophil-mediated amplification of the initial injury.

The existence of such an injury-amplification mechanism appears counterintuitive at first, but could be advantageous for the host in specific setting: by exerting a co-stimulatory signal for the activation of the immune system as suggested in the danger theory by Matzinger [5], HMGB1-mediated neutrophil recruitment could constitute a “preemptive strike” against potential secondary infection, or could constitute a mechanism of immunogenic cell death in the setting of infections that induce necrosis. Of note, the modulation of cell death pathways has been shown to be a major virulence factor for several pathogens, suggesting a central role for cell-death induced immune responses in antimicrobial defense. Further studies are needed to understand how HMGB1 release benefits the host in the setting of acute injury.

Based on our study and previous studies employing pharmacologic HMGB1 inhibition [6], HMGB1 represents a potential target for therapeutic interventions in acute liver injury. Further studies are also needed to determine whether functions of HMGB1 are similar in other organs as in the liver, and could be therapeutically exploited in settings where ischemia-reperfusion plays a key role, such as in organ transplantation. It is also conceivable that HMGB1 may have a role in chronic disease processes that are linked to cell death and chronic inflammation such as atherosclerosis, fibrosis and cancer. In view of previous studies showing a key role of formyl-peptides, another class of DAMPs, in mediating inflammation after heat-induced liver necrosis [7], further research is also required to determine whether DAMPs are injury-, context- and possibly even organ-specific. The sheer number of DAMPs suggests either fine-tuning of host responses by different DAMPs, activation of distinct target cells by different DAMPs, or a high level of redundancy in the immunological sensing of tissue damage. In summary, we need to learn more about “molecular fingerprints” of cell death in order to develop improved therapeutic strategies for cell death-induced inflammation in both acute settings as well as chronic disease such as atherosclerosis and cancer.
